# The emerging role of the branched chain aminotransferases, BCATc and BCATm, for anti-tumor T-cell immunity

**DOI:** 10.1097/IN9.0000000000000014

**Published:** 2023-01-11

**Authors:** Tanner J. Wetzel, Sheila C. Erfan, Elitsa A. Ananieva

**Affiliations:** 1 Department of Biochemistry and Nutrition, Des Moines University, 3200 Grand Avenue, Des Moines, IA, USA

**Keywords:** BCATc, BCATm, BCAA, leucine, immunotherapy, TME

## Abstract

Challenges regarding successful immunotherapy are associated with the heterogeneity of tumors and the complex interactions within the surrounding tumor microenvironment (TME), particularly those between immune and tumor cells. Of interest, T cells receive a myriad of environmental signals to elicit differentiation to effector subtypes, which is accompanied by metabolic reprogramming needed to satisfy the high energy and biosynthetic demands of their activated state. However, T cells are subjected to immunosuppressive signals and areas of oxygen and nutrient depletion in the TME, which causes T-cell exhaustion and helps tumor cells escape immune detection. The cytosolic and mitochondrial branched chain amino transferases, BCATc and BCATm, respectively, are responsible for the first step of the branched chain amino acid (BCAA) degradation, of which, metabolites are shunted into various metabolic processes. In recent years, BCAT isoenzymes have been investigated for their role in a variety of cancers found throughout the body; however, a gap of knowledge exists regarding the role BCAT isoenzymes play within immune cells of the TME. The aim of this review is to summarize recent findings about BCAAs and their catabolism at the BCAT step during T-cell metabolic reprogramming and to discuss the BCAT putative role in the anti-tumor immunity of T cells. Not only does this review acknowledges gaps pertaining to BCAA metabolism in the TME but it also identifies the practical application of BCAA metabolism in T cells in response to cancer and spotlights a potential target for pharmacological intervention.

## 1. Introduction

Progress in cancer immunotherapy raised hopes that cancer patients would experience long-term control or eradication of their disease. However, for many patients, the cancer immunotherapies have been unsuccessful due to substantial drug resistance, side effects, and low remission rates ^[1–5]^. A major obstacle to successful immunotherapy is tumor-evolved mechanisms, which lessen the effectiveness of the anti-tumor T-cell response ^[2,6,7]^. Such mechanisms include, but are not limited to, the release of immunosuppressive signals and areas of nutrient and oxygen depletion, which lead to T-cell exhaustion and inhibition of T-cell function ^[8]^.

Cancer cells exhibit significant control over their microenvironment. This microenvironment is a dynamic and complex network between cancer cells and non-malignant components, such as immune cells, fibroblasts, extracellular matrix, and cytokines ^[9]^. For the cancer growth to be successful, it must remain undetected by the tumor-infiltrating cytotoxic CD8^+^ T cells (CTLs) and the helper type 1 CD4^+^ (T_H_1) cells, while other immune cells, such as myeloid-derived suppressor cells (MDSCs), tumor-associated macrophages M2, and the fork head box protein 3-positive (Foxp3^+^) regulatory T cells (Tregs), must be recruited to dampen the local immune response for improved survival of the cancer cells ^[10,11]^.Years of scientific investigation demonstrated that metabolic enzymes, specialized in breaking down amino acids, such as tryptophan, arginine, phenylalanine, exert immunosuppressive properties in the tumor microenvironment (TME), and are viewed as targets for pharmacological manipulation ^[11]^.

The cytosolic and mitochondrial branched chain amino transferases, BCATc and BCATm, otherwise referred to by gene names *BCAT1* and *BCAT2*, respectively, are responsible for the first step in the degradation of the branched chain amino acids (BCAAs), leucine, isoleucine, and valine. These proteins have only recently emerged as candidates with immunosuppressive properties ^[12–14]^. BCAAs are required for survival of both cancer and immune cells; they are a well-established source of fuel and by-products of their degradation are used to support the increased biosynthetic demands of proliferating cells ^[15]^. A growing body of evidence demonstrated an increased expression of the BCAT isoenzymes in cancers found throughout the body ^[16–18]^, yet significantly less investigations occurred regarding the role of these isoenzymes in the immune cells responsible for tumor clearance. This gap highlights the question about the importance of BCAA metabolism in TME, more specifically, the role these isoenzymes may have in the interactions between immune and tumor cells. Thus, the aim of this review is to discuss the current state of knowledge regarding the role of BCAA catabolism during the T-cell metabolic reprogramming in the TME. Special emphasis is given to the BCAA leucine, and the reversible transamination of leucine by the BCAT isoenzymes. This aligns with the current understanding that leucine is essential for mTORC1 upregulation during T-cell activation. Furthermore, the review discusses what we learned from studies with BCAT deficient T cells or pharmacological targeting of the BCAT step in macrophages and it connects these findings with the proposed function of BCATc and BCATm as new immune metabolic checkpoints in the TME.

## 2. The enhanced bioenergetic demands of activated T cells are dependent on metabolic reprogramming

Cellular metabolism plays a pivotal role in the maintenance of T-cell homeostasis as the plasticity of T-cell metabolism affords them with the flexibility to quickly switch from quiescent to activated state in response to antigen presentation. This feature of T-cell metabolism is commonly referred to as metabolic reprogramming, allowing T cells to meet their increased biosynthetic demands during times of rapid growth and proliferation ^[19]^.

Naive T cells (TN) have basic catabolic needs that afford them with long-term survival in the absence of antigen stimulation. TN cells rely primarily on mitochondrial oxidative phosphorylation and fatty acid oxidation to generate energy during their quiescent stage ^[20]^. Signals from sphingosine-1-phosphate (S1P) suppress mitophagy, allowing the TN cells to maintain mitochondrial content ^[21–23]^.

**Figure 1.**
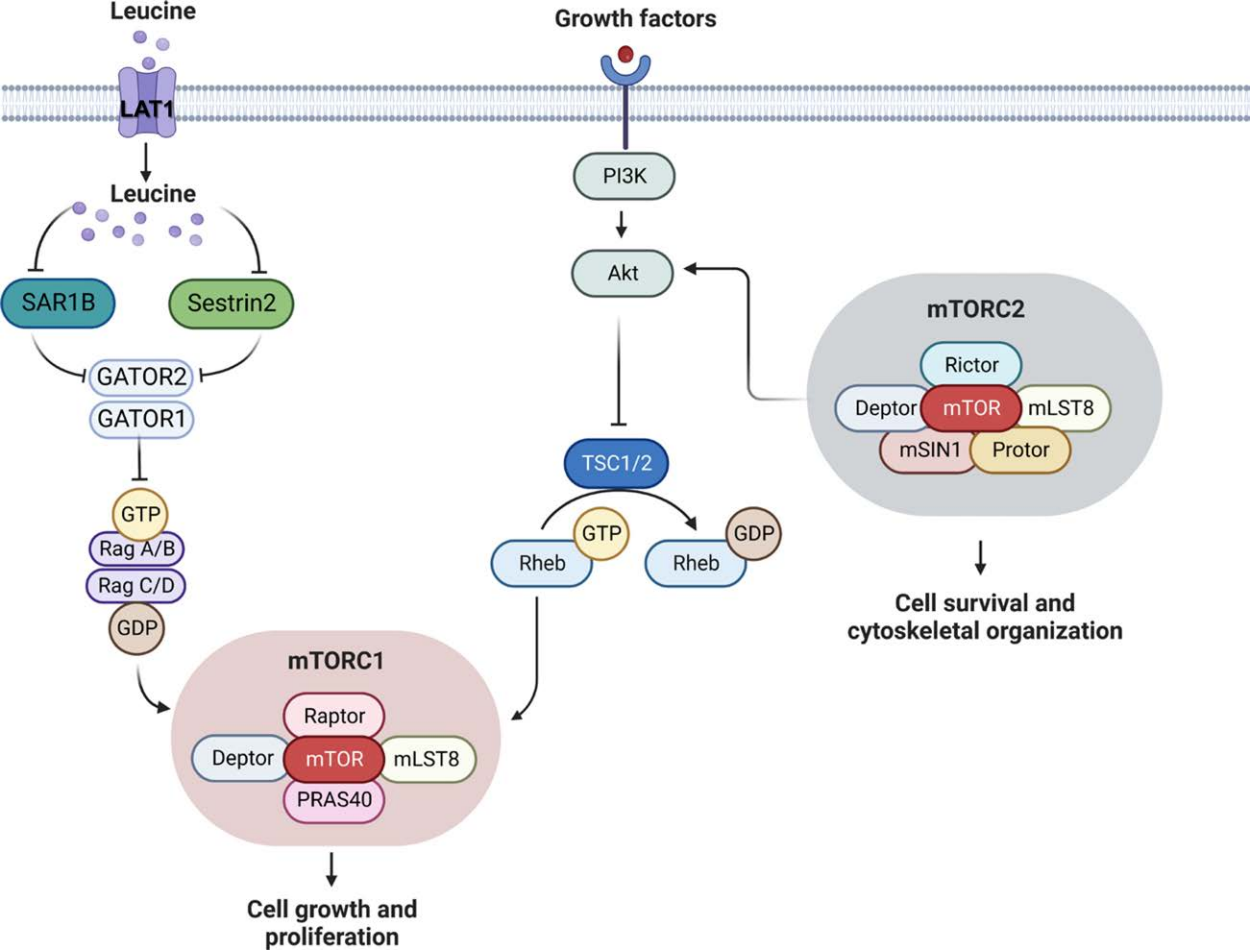
**Overview of mTOR signaling**. The schematics includes the domain structure of mTORC1 and mTORC2 and their main components. Upstream sensing of leucine or growth factors regulate mTORC1 and the transduction of signals that lead to stimulation of cell growth and proliferation. Leucine binds Sestin2 or SAR1B and prevents their interaction with GATOR2 thus releasing the heterodimeric RagA/B-RagC/D GTPases from the inhibitory complex with GATOR1-GATOR2 allowing mTORC1 activation. mLST8: mammalian lethal with Sec3 protein 8; mTORC1 domain: Deptor, DEP-domain-containing mTOR interacting protein; mTORC2 domain: mSIN1: mammalian stress-activated protein kinase interacting; PRAS40: proline rich Akt substrate 40 kDA; Raptor: regulatory-associated protein of mTOR; Rictor: rapamycin-insensitive companion of mTOR; SAR1B: secretion-associated Ras-related GTPase 1B.

A central regulatory switch of T-cell metabolism is the mammalian target of rapamycin (mTOR) that exists as two multi-subunit complexes of regulatory proteins (Figure 1). The rapamycin sensitive complex 1 (mTORC1) is associated with Raptor, while the rapamycin-insensitive complex 2 (mTORC2) is associated with Rictor. Each of these complexes respond to specific sets of environmental stimuli and upstream regulators while transducing signals to different downstream targets ^[24]^. Suppression of the mTORC1 signaling pathway by the upstream tuberous sclerosis complex (TSC) in TN cells allows for the maintenance of quiescence ^[20]^. Removal of TSC from CTLs leads to mTORC1 activation, exit of quiescence, and metabolic reprogramming with increased glycolysis ^[25,26]^. On the other hand, mTORC2 activity supports quiescent-like memory T-cell formation during activation ^[26]^. Thus, suppression of mTOR signaling is critical to maintaining low metabolic activity in quiescent T cells, while activated mTOR signaling rapidly increases metabolic reprogramming and facilitates T-cell transition from quiescence to activation.

Upon antigen presentation on the major histocompatibility complex 1 (MHC1) and binding to the T-cell receptor (TCR), along with costimulatory signals through the CD28 signaling pathway, TN cells differentiate into effector T cells. Following activation, production of environmental cues transduced via upregulated mTORC1 signaling influence further differentiation into CTLs, T_H_1, T_H_2, T_H_17 cells, and Tregs ^[27–29]^. These T-cell subsets have unique roles in immunity ranging from clearance of infections, or destruction of cancer cells (CTLs, T_H_1, TH2), to autoimmunity, inflammation (TH17), and tolerance to maintain immune homeostasis (Tregs) ^[30]^. While all subtypes undergo metabolic reprogramming, the unique direction in which these subtypes regulate the immune response comes with different metabolic preferences when they reach terminal differentiation. For the purposes of this review, T_H_2 cells will be excluded as they do not pertain to the cell-mediated immune response.

Activated CTLs, TH1, and TH17 depend heavily on glycolysis and amino acid metabolism but less on oxidative phosphorylation ^[31–34]^. The reliance on glycolysis is supported with activation of mTORC1, leading to upregulation of the glucose transporter (Glut1) and concomitant influx of glucose into the cells ^[35]^. Upregulation of amino acid transporters, such as solute carriers Slc7a5-Slc3a2, Slc1a5, and Slc38a2 allows influx of BCAAs, glutamine, and alanine, which are used for protein and nucleotide synthesis during rapid phase of growth of the CD4^+^T_H_1 and CTL cells ^[36,37]^.

**Figure 2.**
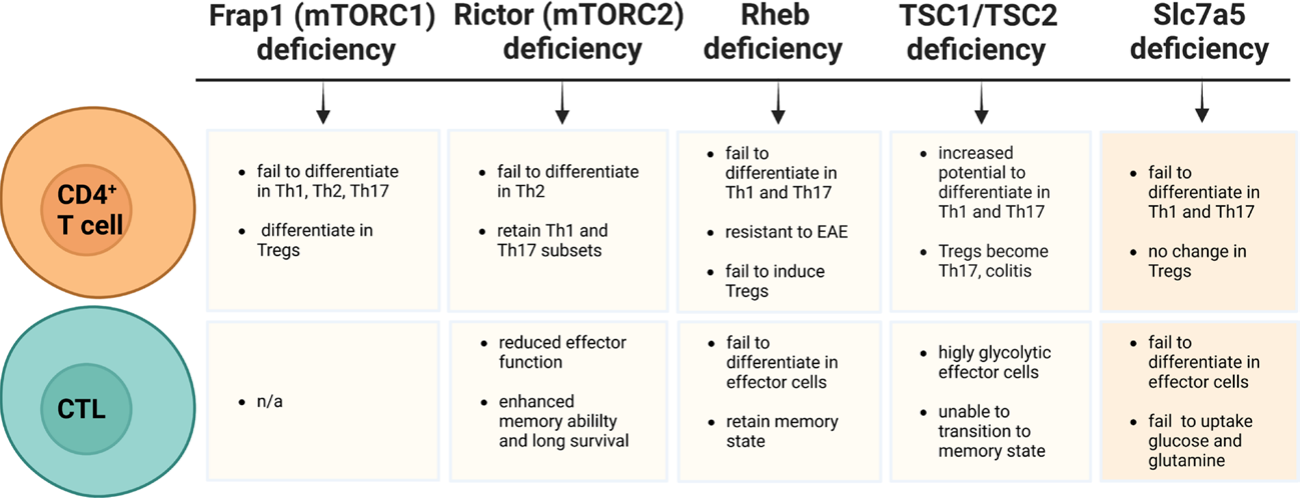
**Summary of the effects of genetic perturbation of mTOR signaling and LAT1 in T cells**. The schematics compares the effects of deleting major mTOR components and the Slc7a5 subunit of the LAT1 transporter on the differentiation and lineage commitment of CD4^+^ T cells and CTLs. The schematics is based on findings reported in mice with T cells deficient in: (1) Frap1 ^[38]^, (2) Rictor, Rheb, or TSC2 ^[26]^, (3) TSC1 ^[39]^, or (4) Slc7a5 ^[37]^. CTLs: cytotoxic CD8^+^ T cells; mTOR: mammalian target of rapamycin; TSC: tuberous sclerosis complex.

The central role of mTORC1 in orchestrating the metabolic reprogramming of CD4^+^ T cells and CTLs is well studied in mice with T-cell-specific deletions of main components of the mTORC1 and mTORC2 signaling pathways (Figure 2) ^[26,38,40]^. Deletion of TSC2 from T cells results in the generation of highly glycolytic CTLs incapable of transitioning into memory T cells. In contrast, CTLs deficient in the RAS homolog enriched in brain (Rheb) fail to differentiate into effector T_H_1 and TH17 cells but retain their memory features, including low metabolic rate and increased longevity ^[41]^. Rheb is an upstream activator of Raptor, which is active when TSC1/TSC2 complex is inhibited (Figure 1) ^[42]^. Inhibition of mTORC2 by deletion of Rictor in CD4^+^ T cells impacts the ability of these cells to differentiate in T_H_2, but not in T_H_1 or TH17 subtypes. In contrast, inhibition of mTORC1 by deletion of the gene encoding mTOR (*Frap1*) in mouse CD4^+^ T cells makes them unable to differentiate into T_H_1, T_H_2, TH17 or to produce interferon-gamma (IFNγ). Instead, they differentiate into Tregs (Figure 2) ^[43]^. Moreover, simultaneous inhibition of both complexes, mTORC1 and mTORC2, causes potent induction of Tregs in the absence of exogenous transforming growth factor beta (TGF-β) ^[41]^. Thus, Tregs are stimulated under conditions that downregulate mTOR signaling, and in contrast to CTL, T_H_1, and T_H_17 cells, Tregs demonstrate an increase in reliance on fatty acid oxidation and oxidative phosphorylation but not on glycolysis ^[31,33,44–46]^. This suggests that Tregs function relates to a long-term, sustainable energy sources instead of the need for rapid energy production. Glycolysis may play a role in early Treg differentiation, but long-term reliance may suppress survival and lineage commitment of these cells ^[47]^.

In summary, T-cell fate is dependent on signals that are transduced via mTOR, which drives T-cell differentiation and function. Powell and Delgoffe (2010) proposed a model where high mTOR activity parallels a state of high T-cell activation, favoring the generation of T_H_1 and CTLs, which develops during pathogen clearance or cancer cell eradication ^[1,41]^. Alternatively, as the immune response is no longer needed or must be suppressed, T cells experience decreased mTOR activation and differentiation to Tregs or CTL memory subtypes are favored. This transition is modulated by nutrients, of which, the BCAA leucine is one of the most potent nutrient activators of mTORC1 and is discussed below.

## 3. The role of BCAAs and BCAA transport in T-cell metabolic reprogramming

Being essential amino acids, the BCAAs are internalized using specialized transporters, which ensure delivery of these amino acids for vital processes inside the cell. Apart from being incorporated in newly synthesized proteins, the BCAAs, and particularly leucine, serve as potent nutrient activators of mTORC1 signaling ^[31,37,48]^. The most recent understanding of the mechanistic detail of this activation includes GATOR1, GATOR2, Sestrin2, and SAR1B ^[49,50]^. GATOR1 and GATOR2 transduce signals to mTORC1 signaling by sensing amino acid deficiency (GATOR1) or sufficiency (GATOR2). Upon interaction with GATOR2, Sestrin2 inhibits mTORC1 signaling. However, binding of leucine to Sestrin2 prevents the GATOR2-Sestrin2 interaction leading to mTORC1 activation (Figure 1) ^[49]^. SAR1B is a small GTPase that was recently discovered by Chen and co-authors (2021) to serve as another upstream regulator of mTORC1 that directly binds GATOR2 and leucine (Figure 1) ^[50]^. However, the interaction of SAR1B with leucine differs from that of Sestrin2 with leucine in that they have different sensitivity to leucine and recognize different structural features of leucine ^[50]^. Sestrin2 belongs to a family of proteins that can be induced by DNA damage and nutritional stress to inhibit mTORC1 when conditions are not favorable for growth ^[51]^. SAR1B is involved in the transport of pre-chylomicrons from the endoplasmic reticulum to the Golgi. Rare genetic defects in the SAR1B gene manifest in fat malabsorption and fat-soluble vitamin deficiency ^[52]^.

While little is known about Sestrin2 and SAR1B in T cells, their discovery as leucine sensors further supports the important role of leucine as a nutrient switch of mTORC1 signaling. Whether leucine is sensed by Sestrin2 or SAR1B in activated T cells is yet to be discovered, but depletion of leucine or defective leucine transport in T cells can impact mTORC1 signaling in T cells and inhibit T-cell activation. Jurkat T cells, or previously activated 5CC7 T_H_1 cells, stimulated with varying concentrations of *N*-acetyl-leucine amide (NALA), a structural analog of leucine, fail to upregulate mTORC1 while secreting reduced amounts of IFNγ and interleukin 2 in response to co-stimulation with anti-CD3 and anti-CD28 ^[53–55]^.

Leucine, isoleucine, and valine transport across the cell membranes requires an L-type amino acid transporter (LAT). The LAT family is represented by several members, one of which is LAT1 (Slc7a5) that forms a heterodimer with the transmembrane protein Slc3a2 ^[48,56]^. Mutant T cells deficient in either Slc7a5 or Slc3a2 were studied in mice, and, in all cases, the defective phenotype of the resulting T cells is partially explained by interruption of BCAA uptake and impact on mTORC1 signaling ^[37,57]^.

Deletion of the gene encoding the Slc7a5 transporter prevents the proliferation and differentiation of mouse T_H_1, T_H_17, and CTLs but not that of Tregs cells. Slc7a5-null T cells fail to upregulate glucose uptake or switch to glycolysis (Figure 2). Sinclair and co-authors ^[37]^ explain the T-cell metabolic catastrophe in part by the failure of the Slc7a5-null T cells to sustain leucine uptake for mTORC1 activity. In another study, mice deficient of Slc3a2 in Foxp3^+^ Tregs show significantly reduced isoleucine uptake. These mice have reduced numbers and reduced proliferation of Foxp3^+^ but not Foxp3^-^ Tregs and are unable to suppress intestinal inflammation. This study emphasizes on the importance of isoleucine, but not leucine or valine, for the maintenance of the proliferative state of Tregs after their lineage commitment ^[57]^. In another study, the expression of Foxp3 in Tregs is increased in the presence of low leucine, a finding consistent with Ananieva’s unpublished data that treatment with NALA upregulates the expression of Foxp3 in activated mouse CD4^+^CD25^+^ T cells ^[58]^. A follow-up study with activated human T cells further confirms that T cells experience an increased influx of BCAAs via LAT1 during T-cell activation ^[59]^.

Leucine transport via LAT1, however, is dependent on the presence of glutamine. Glutamine enters the cells via a separate transporter, Slc1a5, while LAT1 uses intracellular glutamine as an efflux substrate to regulate the uptake of extracellular leucine into the cells. The knockdown of either transporter via SiRNA in HeLa cells inhibits mTOR activation, suggesting each is an important step in mTOR activation ^[36,60]^. This is further confirmed by pretreatment of HeLa cells with glutamine that leads to a rapid increase in leucine uptake and activation of the mTORC1 downstream target, the 70 kDA ribosomal protein S6 kinases 1 and 2 (S6K-1 and 2). Importantly, it is shown that glutamic acid and α-ketoglutarate do not regulate S6K-1 ^[60]^. While these studies used HeLa cells as a model system, a study by Carr and co-authors ^[36]^ showed that T cell activation requires a large increase in glutamine uptake assisted by additional glutamine transporters, such as the sodium-coupled neutral amino acid transporters, SNAT1 and SNAT2. Depletion of glutamine, on the other hand, blocks T cell proliferation and IFNγ secretion, and cannot be rescued by other amino acids, such as glutamate ^[36]^.

Ultimately, this discussion highlights the uptake of BCAAs and glutamine in activated T cells as a critical component of their metabolic reprogramming, which may exist to ensure proper T-cell function and expansion.

## 4. Transamination is the most important step of BCAA degradation exploited by activated T cells

**Figure 3.**
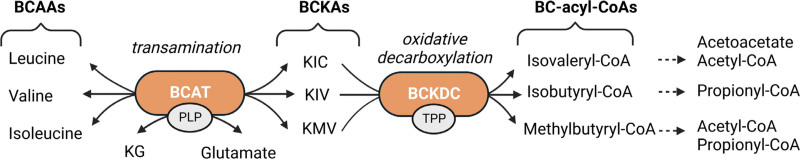
**Simplified representation of the BCAA degradation pathway**. The first two steps in BCAA degradation are shown. Step 1 is a freely reversible transamination catalyzed by BCATc or BCATm and step 2 is the irreversible oxidative decarboxylation catalyzed by the BCKDC complex. Following step 2, each BCAA commits to their own degradation pathway, leading to the formation of common metabolic intermediates, such as acetyl-CoA, acetoacetate, or propionyl-CoA. BCAAs: branched chain amino acids; BC-acyl-CoAs: branched chain acyl CoAs; BCKAs: branched chain keto acids; KG: α-ketoglutarate; KIC: α-ketoisocaproic acid; KIV: α-ketoisovaleric acid; KMV: α-keto- β-methylvaleric acid; PLP: pyridoxal phosphate (cofactor for BCAT); TPP: thiamine pyrophosphate (cofactor for BCKDC).

Around 20% of the BCAAs entering the cells are subjected to degradation. The first step in BCAA degradation is the reversable transamination catalyzed by BCATc and BCATm isoenzymes. This reaction comprises a transfer of the α-amino group of a BCAA to α-ketoglutarate (α-KG) to form glutamate (Glu) and the respective branched chain α-keto acid (BCKA) (Figure 3) ^[48,61,62]^. For the BCAAs to commit to their unique degradation pathways, the BCKAs must be subjected to irreversible oxidative decarboxylation by the mitochondrial branched chain α-keto acid dehydrogenase complex (BCKDC), which exists as a multienzyme complex organized around a core scaffold of dihydrolipoamide acyltransferase (E2) to which the branched chain α-keto acid dehydrogenase (E1) and the dihydrolipoamide dehydrogenase (E3) are attached. After this step, each BCAA commits to their catabolic pathway with final products being acetyl-CoA, acetoacetate, or propionyl-CoA (Figure 3) ^[63]^. Of those final products of BCAA degradation, acetyl-CoA is a vital molecule for sustaining normal cellular function, providing acetyl groups required for acetylation and gene activation ^[64]^. It has been shown that in the context of bacterial infection, small chain fatty acids (SCFAs) metabolized to acetyl-CoA contribute to the upregulation of mTOR activity in T cells ^[64]^. Secondly, decreased acetyl-CoA production due to the knockout of the peroxisomal acyl-coenzyme A oxidase 1 (ACOX1) in hepatocytes, a rate-limiting enzyme in β-oxidation of fatty acids, decreases Raptor acetylation and reduces lysosomal localization of mTORC1, leading to reduced mTOR activity. Rescue of the hepatocyte-ACOX1^KO^ mouse with dichloroacetic acid replenishes acetyl-CoA levels and restores mTORC1 activity in hepatocytes ^[65]^. In addition, knocking down 3-methylcrotonyl-CoA carboxylase 1 (MCCC1), an enzyme involved in the leucine degradation pathway downstream of BCAT, decreases acetyl-CoA concentrations and reduces mTOR activity in HeLa cells ^[66]^. Taken together, these findings suggest acetyl-CoA may play a role in mTOR activation. However, the significance of these studies in the context of BCAA metabolism in T cells is not very clear. As discussed below, activated T cells commit only 2%–3% of BCAAs to irreversible oxidation, while the majority of BCAAs are re-cycled or used for protein synthesis. Thus, it is unlikely that BCAA degradation is a significant source of acetyl-CoA, acetoacetate, or propionyl-CoA in activated T cells.

BCATm and BCKDC are ubiquitously expressed in most adult human tissues, and they work in tandem in the mitochondria to afford the cells with transamination and oxidation of the BCAAs ^[48]^. In contrast, BCATc is responsible for the cytosolic transamination of BCAAs and is mostly expressed in the central nervous system and proliferating cells ^[18,67]^.

**Figure 4.**
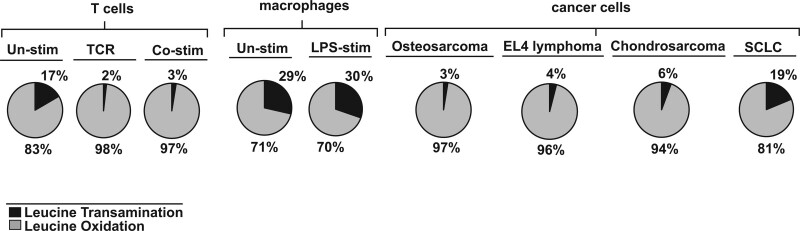
**Comparison of leucine catabolism in immune and cancer cells**. The two reactions in leucine catabolism, transamination, and irreversible oxidation, were compared as a percent distribution in T cells, microphages, and cancer cells based on unpublished and published reports ^[54,69,70]^. co-Stim: anti-CD3 and anti-CD28 co-stimulated mouse CD4^+^ T cells; EL4: murine T-cell lymphoma; LPS: lipopolysaccharide stimulated Raw 264.7 macrophages; SCLC: murine small-cell lung cancer; TCR, anti-CD3 stimulated CD4^+^ mouse T cells; un-stim: unstimulated murine CD4^+^ T cells or Raw 264.7 macrophages.

As published by Ananieva and co-authors in 2014, the transamination of BCAAs in naïve CD4^+^ T cells occurs solely in the mitochondria where BCATm and BCKDC are located. As early as 8 hours following TCR receptor activation, the total BCAT activity increases over that of naive T cells. This increase in BCAT activity is attributed to BCATc, which is upregulated in response to TCR engagement. Consistent with the change in BCATc expression, leucine transamination is increased ^[48,54]^. In contrast, leucine oxidation is reduced consistent with the metabolic switch of the activated T cells from catabolic to anabolic processes that demand the preservation of the amino acid carbon skeletons for building purposes, while oxidative processes are inhibited ^[48,68]^. Comparison of leucine transamination and oxidation, based on findings by Ananieva and co-authors (2014) (Figure 4), indicates that naïve T cells oxidize around 17% of leucine, while activated CD4^+^ T cells slow down this process to around 3% ^[54]^. The cancerous counterpart of mouse T cells, EL4 lymphoma, appears to oxidize ~4% of available leucine. In contrast, other cancer cells (chondrosarcoma, small lung cancer) or innate immune cells (macrophages) retain better ability to oxidize leucine suggesting that the leucine degradation pathway may be one of the preferred sources of fuel ^[69]^. Interestingly, mouse-derived macrophages oxidize around 20% of leucine regardless of whether they are lipopolysaccharide (LPS)-stimulated (Figure 4). Macrophage utilization of leucine oxidation during their polarization into anti-inflammatory M1 or the pro-inflammatory M2 types is yet to be investigated, but studies focusing on the BCATc isoenzyme in human primary macrophages suggest a role for BCATc in the metabolic events leading to the broken (fragmented) tricarboxylic acid (TCA) cycle ^[71,72]^. Inhibition of BCATc by the leucine analog ERG240 suppresses production of the immune-responsive gene (IRG1) and itaconate in LPS-activated macrophages. Silencing of the BCATc gene in those cells resembles the effect of the pharmacological blockade with ERG240 resulting in reduction of oxygen consumption and glycolysis along with decreased IRG1 and itaconate levels ^[72]^. Further investigation, however, reveals that the uptake of BCAAs during the early activation of the human macrophages is not increased and therefore not considered essential for the LPS driven fragmented TCA cycle, implicating an alternative function of BCATc beyond its catalytic activity ^[73]^. In contrast, a recent study with macrophages (THP-1 cells) and primary human monocytes treated with 1α,25-dihydroxyvitamin D3 reveals increased levels of BCATc and BCKDC along with downregulation of mTORC1 signaling and enhanced autophagy ^[13]^. Taken together these findings imply that: (1) BCAA catabolism might contribute to the metabolic reprogramming of macrophages in time and context dependent manner and (2) the preferences for BCAA catabolism of T cells and other immune cells, such as macrophages, may depend on their unique patterns of differentiation or polarization and specificity of immune function.

## 5. Studies with T cells deficient in BCATc and BCATm may shed further light on the immunosuppressive potential of these enzymes

Activated CD4^+^ T cells isolated from the global BCATcKO mice have been shown to transaminate less leucine compared to similarly activated wildtype T cells, leading to an increase in the intracellular leucine concentrations and higher activity of mTORC1 signaling, which suggests a link between BCAA metabolism and mTORC1 activation in the development of the T-cell immune response ^[54]^. Indeed, BCATc-deficient CD4^+^ T cells have enhanced glycolysis, higher glycolytic capacity, improved oxygen consumption, and increased capacity to synthesize ATP along with increased secretion of IFNγ. The leucine structural analog NALA suppresses mTORC1 activity and IFNγ secretion, while the inhibitor of the BCATc isoenzyme activity, gabapentin, has the opposite effects ^[48,54]^. These findings indicate that active BCATc isoenzyme must be present to exert the observed effects. While BCATc is described as important to control the large cytosolic influx of leucine following T-cell activation, the role of BCATm does not appear as critical since BCATm expression is not upregulated upon T-cell activation ^[54]^. Published ^[48]^ and ongoing research in the Ananieva laboratory further shows that CD4^+^ T cells deficient in BCATm are able to upregulate mTORC1 signaling, secrete more IFNγ, and improve their glycolytic and oxygen metabolism over that of T cells expressing normal levels of BCATm. This implies that mitochondrial transamination of leucine still occurs and BCATc alone is not sufficient to regulate the increased influx of leucine in response to T-cell activation.

**Figure 5.**
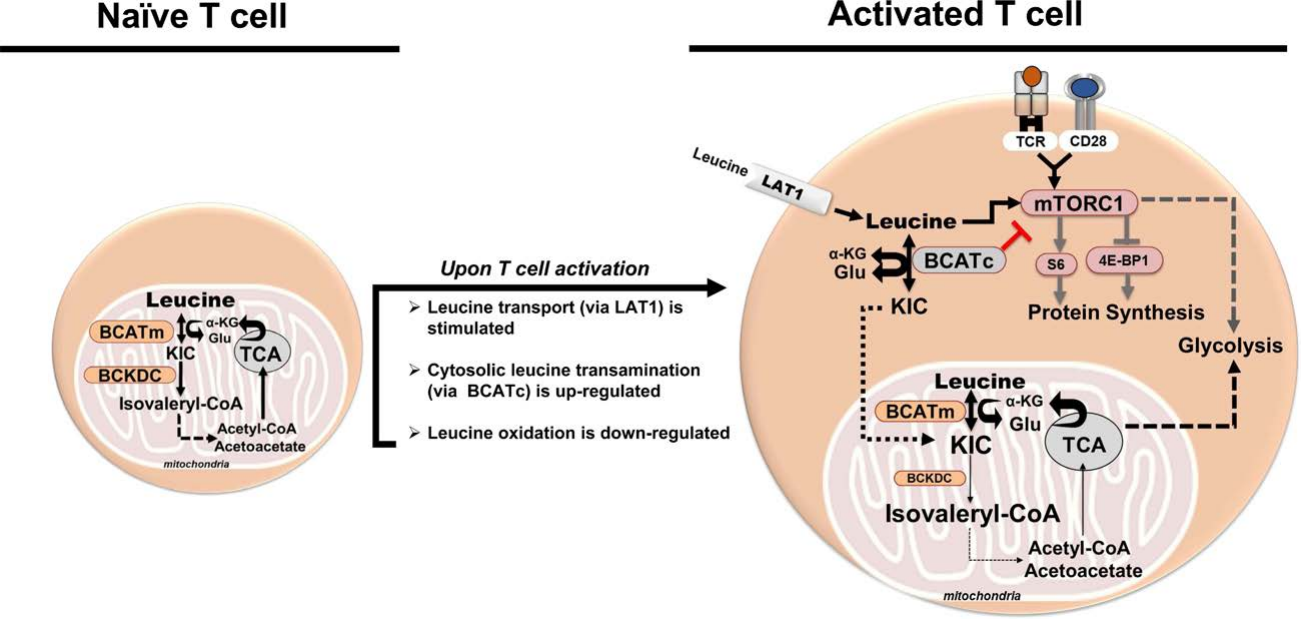
**Model of BCAT regulation of T-cell activation. Activated T cells experience increased uptake of leucine assisted by the LAT1 transporter**. Leucine activates mTORC1, which stimulates protein synthesis and glycolysis leading to T-cell activation. The gene encoding BCATc is induced via the TCR signaling (not shown). BCATc initiates cytosolic transamination of leucine providing a negative feedback regulation of mTORC1 activity. This model is based on findings with activated mouse CD4^+^T_H_1 cells reported by Ananieva et al ^[54]^. 4E-BP1: eukaryotic translation initiation factor 4E-binding protein 1; α-KG: α-ketoglutarate; BCAT: branched chain aminotransferase; BCKDC: branched chain α-keto acid dehydrogenase complex; CD28: cluster of differentiation 28 that provides the costimulatory signal for T-cell activation; Glu: glutamate; KIC: α-ketoisocaproate; mTORC1: complex 1 of mTOR; S6: ribosomal S6 protein; TCR: T-cell receptor.

A model of BCAT regulation of T-cell function can be proposed based on the key studies done with T cells deficient in leucine transporters, or the BCAA metabolizing enzymes BCATc, and BCATm ^[37,48,54]^. According to this model, naive T cells, which are primarily catabolic, use mitochondrial leucine metabolism to meet basic metabolic needs. Upon TCR engagement and subsequent T-cell activation, leucine transport via Slc7a5 is stimulated, while BCATc expression is upregulated allowing cytosolic leucine transamination to occur. By using leucine as a substrate for its transamination reaction, BCATc limits leucine availability to mTORC1 signaling. BCATc alone is not sufficient to maintain this negative feedback loop as BCATm appears important to sustain mitochondrial leucine transamination during T-cell activation (Figure 5).

## 6. BCATc and BCATm are new candidates for immune metabolic checkpoints in the TME

The efficacy of the cancer immunotherapies is limited to a narrow subset of patients with a low percentage of remission rates ^[3,74]^. A critical barrier for the success of these therapies is the existence of immunosuppressive signals in the TME ^[7]^. The heterogeneous tumor mass elicits a variety of immunosuppressive signals originating from tumor cells and tumor-promoting immune cells, such as tumor-associated M2 macrophages, MDSCs, or Tregs ^[11]^. The immunosuppressive signals range from cytokines, such as TGF-β, interleukin-10 and interleukin 35, reactive oxygen species and metabolic enzymes. When the tumor-eradicating T cells, such as the CTLs, T_H_1, and TH17, infiltrate the TME, they become subjected to immunosuppressive signals, but also to areas of oxygen and nutrient depletion, which ultimately leads to their exhaustion. The robustly growing tumors take advantage of the immunosuppressive signals while reprogramming their metabolism to adapt to high influx of glucose and amino acids and upregulate anabolic processes needed for further growth and proliferation ^[75]^. The high glycolytic activity of the tumor cells causes areas of low oxygen, buildup of lactate and concomitant acidification of TME. These changes influence how CTLs recognize and eliminate tumor cells allowing the growing tumors to escape immune destruction. CTLs and TH1 are less adaptive to the tumor-imposed changes in TME, which makes them less efficient in competing for nutrients and exerting their effector functions ^[11]^.

Among the amino acids, arginine and tryptophan, and associated metabolic enzymes, most notably received scientific attention regarding the impact these amino acids and their degradation pathways have on T-cell function and survival in the TME. Arginine and tryptophan are essential for T cells as their depletion from the local microenvironment results in T cell cycle arrest. Indoleamine 2, 3-dioxygenase (IDO) and tryptophan-2,3-dioxygenase (TDO), the enzymes catalyzing the rate-limiting step in tryptophan degradation, can cause local depletion of tryptophan leading to the inhibition of T_H_17 differentiation and T-cell apoptosis. Similarly, arginase 1, which is responsible for the conversion of arginine into ornithine and urea, is a well described immunosuppressive enzyme that limits the availability of arginine to CTLs. Altogether these enzymes have immunomodulatory functions during tumorigenesis and have been targeted for pharmaceutical interventions in clinical trials ^[48,76,77]^.

Most recent studies demonstrate that glutamine also plays a role within the TME as it serves as a carbon and nitrogen source for numerous metabolic pathways, including, but not limited to, nucleotide synthesis, amino acid production, extracellular matrix production, and redox balance ^[78]^. Suppression of glutamine uptake in the TME by using the competitive antagonist of glutamine influx, V9302, leads to a reduction in tumor mass and decreases T-cell infiltration but increases the presence of M2-type macrophages ^[79]^.

Similar to arginine and tryptophan metabolism, glutamine metabolism in TME promotes cancer cell growth while impairing anti-tumor T-cell immunity. Blockade of glutamine metabolism by the use of the glutaminase antagonist, JH083, suppresses the metabolic reprogramming of cancer cells but enhances the anti-tumor CD8^+^ T cells and increases tumor infiltration ^[80]^. Inhibition of glutamine metabolism also stimulates the antigen presentation by macrophages to CD8^+^ T cells. Finally, comprehensive inhibition of glutamine metabolism by 6-diazo-5-oxo-norleucine (DON) inhibits the generation of MDSCs and promotes the generation of long-lived and highly activated effector T cells with markedly upregulated oxidative metabolism ^[78,80]^.

The BCAT isoenzymes attracted the attention of oncologists due to their overexpression in cancers and potential to serve as prognostic cancer markers (reviewed in Ananieva and Wilkinson ^[81]^). The initial enthusiasm about BCAT isoenzymes in cancer growth, however, was not associated with exploring their potential immunosuppressive role, which to this day remains poorly understood.

Some published data and ongoing research suggest that the BCAT isoenzymes may exert immunosuppressive functions in the TME ^[12–14,48,54,70]^. As described in Ananieva’s reports, the BCAT isoenzymes might be a part of a critical resistance mechanism that involves a BCAT-dependent negative feedback regulation of mTORC1 signaling during T-cell activation and may serve as an essential metabolic checkpoint of T-cell function in TME ^[48,54]^. In addition, BCAT isoenzymes might be a part of the repertoire of other immune cells of the TME, such as macrophages or dendritic cells ^[82]^.

**Figure 6.**
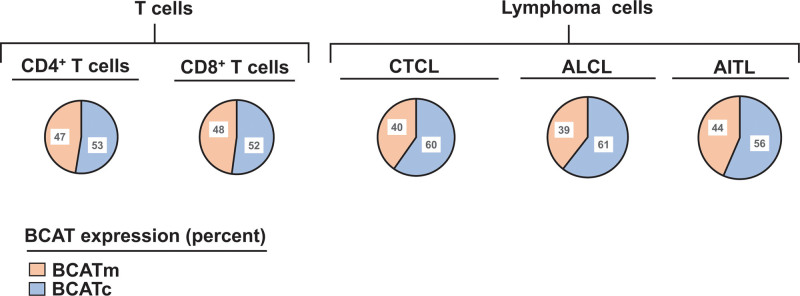
**Comparison of BCATc and BCATm expression in immune and cancer cells**. The expression of each isoenzyme, BCATc and BCATm, was compared between T cells and cancer cells based on a previously published gene profiling study ^[83]^. This information was accessed via the R2 genomics analysis and visualization platform (http://r2.amc.nl). AITL: angioimmunoblastic T-cell lymphoma; ALCL: anaplastic large-cell lymphoma; CTCL: cutaneous T-cell lymphoma.

Gene expression profiling of activated CTL and CD4^+^ T cells, as well as isolated tumor cells from T-cell–derived lymphomas, reveals that BCATc constitutes ~50% of the total BCAT expression in the non-malignant T cells, but this increases to ~60% in cutaneous T-cell lymphoma (CTLC), anaplastic large-cell lymphoma (ALCL) and angioimmunoblastic T-cell lymphoma (AITL). The expression of BCATm is downregulated in those cancerous tissues compared to non-malignant T cells (Figure 6) ^[83]^. Similar trend is observed in another gene profiling study with BCATc being highly expressed in ALCL and angioimmunoblastic T-cell lymphoma (AITL) ^[84]^ suggesting that BCATc may be the preferable isoform in the TME. Consistent with this finding, Li and co-authors (2021) reported a positive correlation between the expression of the BCATc gene and the infiltration levels of CTLs, CD4^+^ T cells, neutrophils, macrophages, and dendritic cells in colon adenocarcinoma, rectum adenocarcinoma, and neck squamous cell carcinoma ^[82]^.

In patients with glioblastoma, the most aggressive primary brain tumor, the gene encoding BCATc is overexpressed in wildtype but not in isocitrate dehydrogenase 1 (IDH1) mutant type forms ^[18]^. The immunological profile of the glioblastoma microenvironment differs between wildtype and IDH1 mutant forms. In IDH1 wildtype glioblastomas, the TME is more immunosuppressive, where Tregs and tumor-associated macrophages contribute to the release of immunosuppressive signals ^[85,86]^. Based on Pearson correlation analysis, the expression of the BCATc gene positively associates with M2 macrophage and Treg markers. This suggests that BCATc is important for the immune signature of aggressive wildtype glioblastoma ^[14]^. In an RNA-sequencing study, focused on the subventricular zone of glioblastomas, the BCATc, but not the BCATm, gene is included in a six-gene signature of patients with high-risk glioblastoma and poor overall patient survival. Those six genes negatively correlate with activated natural killer (NK) cells and monocytes, but positively associate with macrophages and activated dendritic cells and higher program cell death ligand1 (PD-L1) mRNA expression in glioblastoma tissues ^[87]^.

Mechanistic detail regarding the possible role of BCATc and BCAA metabolism in the TME of glioblastoma reveals that glioblastoma cells, expressing high levels of BCATc, excrete significant amounts of BCKAs via the monocarboxylate transporter 1 (MCT1), which is located near the BCATc enzyme ^[85]^. The extracellular BCKAs are taken by M1 macrophages, which convert BCKAs back to BCAAs. M1 macrophages are known to produce T_H_1-type cytokines and participate in phagocyte-dependent inflammation and thus possess anti-tumor potential ^[88]^. The increased uptake of BCKAs reduces the ability of the M1 macrophages to perform phagocytosis. Thus, tumor-derived BCKAs via BCATc may exert the immunosuppressive effect on the glioblastoma TME ^[85]^.

Tumor oxygenation is commonly disturbed in glioblastomas resulting in hypoxia that activates the hypoxia-inducible factor, HIF1-α, which in turn supports glioblastoma progression and invasiveness ^[89]^. In glioblastoma cells, both HIF1 and its partner HIF2 can induce expression of LAT1 causing an increased uptake of BCAAs. Under these conditions, HIF1 upregulates BCATc, but not BCATm, supporting BCAA metabolism via the cytosolic transaminase ^[90]^. Another transaminase, glutamate pyruvate transaminase (GPT, also known as alanine aminotransferase), responsible for the reversible conversion of glutamate into α-KG, requires HIF2, but not HIF1, for expression in human glioblastoma cells ^[91]^. GPT, along with other enzymes, such as glutamate-oxaloacetate transaminase (GOT) and IDH can all produce α-KG that can be used in the TCA cycle, but also as a substrate of the BCATc enzyme reaction. While investigation regarding the status of the BCATc enzyme reaction under hypoxic TME conditions is currently lacking, the mechanism of silencing of BCATc in IDH1 mutant type glioblastoma includes a depletion of α-KG and extensive DNA hypermethylation of the main promoter region of the gene encoding BCATc in IDH1 mutant type of glioblastoma ^[18]^. Interestingly, treatment of naive T cells with cell-permeable α-KG under Treg polarizing conditions significantly reduces the Foxp3^+^ T reg differentiation and this change is associated with altered epigenetic profile of these cells caused by α-KG-triggered DNA methylation. Instead of becoming Foxp3^+^ Treg cells, these cells polarize toward the T_H_1 subset and demonstrate enhanced ability to infiltrate tumor- bearing mice and produce more IFNγ, TNF, and interleukin 17A. On metabolic level, these cells have upregulated mitochondrial metabolism, increased oxidative phosphorylation and increased triglyceride synthesis ^[18]^. While the role of BCATc in Treg cells is yet to be fully characterized, it is plausible to speculate that α-KG might play a role in the epigenetic modification of the gene encoding BCATc in these cells.

In contrast to the increasing interest of BCATc in immunity, the only study that provides experimental findings regarding the role of BCATm in immune cells dates to 2014 where BCATm is described as a constitutively expressed transaminase supporting mitochondrial BCAA catabolism in CD4^+^ T cells ^[54]^. Oppositely, there is a well-established experimental evidence on BCATm being expressed in cancers of different origins and its role as a prognostic cancer marker ^[16,17,70]^. A report by Conway and co-authors ^[92]^ shows that the protein expression of BCATm, along with that of BCATc, is increased in human specimens isolated from IDH-WT gliomas compared to normal brain tissues. Moreover, the authors further demonstrate a significant increase in the protein expression of BCATc and BCATm in IDH1-WT compared to IDH1-mutant glioblastomas ^[92]^.

Studies with pancreatic ductal adenocarcinoma (PDAC) and non-small-cell lung cancer (NSCLC), point toward the importance of BCATm for nucleotide biosynthesis during tumor growth. In PDAC tissues, the expression of BCATm is dependent on malic enzyme 3 (ME3), AMP-dependent kinase (AMPK) and sterol regulatory element binding protien1 (SREBP1). Overexpressed BCATm catalyzes the conversion of BCAAs to BCKAs, during which reaction, glutamate is regenerated and used to support de novo nucleotide synthesis via the glutamate-glutamine axis ^[17]^. In a follow-up study, high BCAA diet stabilizes BCATm necessary to enhance BCAA utilization leading to PDAC progression ^[93]^. In NSCLC tissues, BCATm is overexpressed, where it transaminates BCAAs to BCKAs and provides nitrogen for the biosynthetic processes in the lung cancer cells. Oxidation of BCAAs is reduced in these tissues further supporting the notion that BCAA carbon skeletons and nitrogen are re-cycled during high biosynthetic demands ^[16]^. Lastly, a global deletion of BCATm from C57BL/6 mice results in 10- to 20-fold elevation of BCAAs in the serum and lymphoma tumors developed in these mice. Interestingly, the highly elevated BCAA levels and the blockage of BCAA metabolism at the BCATm step correlate with decreased amounts of glutamine and alanine and reduced tumor burden. This loss of BCATm is associated with a toxic buildup of BCAAs. Thus, perturbation in BCAA catabolism not only depletes the cells of energy but may also interfere with the influx of other amino acids or nutrients needed to support lymphoma growth ^[70]^.

**Figure 7.**
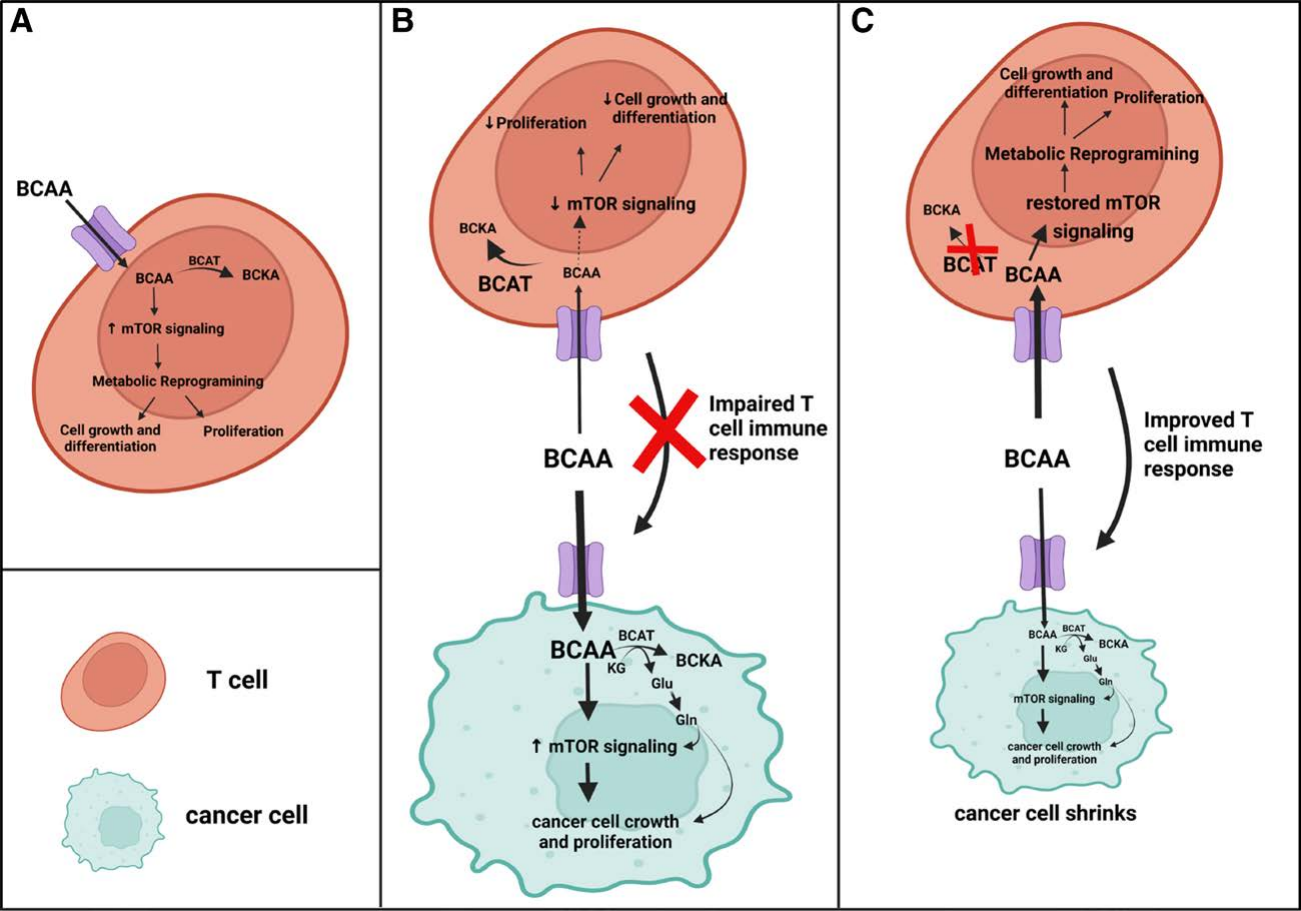
**Comparison of normal, pathologic, and proposed involvement of the BCAT enzymes in T-cell metabolism**. (A) In normal conditions, BCAAs are transported into T cells, leading to an increase in mTOR activation and metabolic reprogramming. This metabolic reprogramming helps fuel T-cell growth, differentiation, and proliferation. BCATs serve as a part of a feedback loop regulation of the BCAA influx. (B) Within the tumor microenvironment, cancer cells express aberrant BCAT activity that sustains cancer growth via the glutamate-glutamine axis. Likewise, the cancer cells uptake large amounts of BCAAs creating a nutrient depleted environment for the T cells. This decreases mTOR signaling and reduces the growth and proliferation of the T cells causing impaired immune response against the cancer cells. (C) Under a proposed model of BCAT removal from T cells, the influx of BCAAs restores mTOR activity, metabolic reprogramming, cell differentiation, and growth. This restoration ultimately improves the immune response of T cells and shrinks tumor growth. BCAA: branched chain amino acid; BCAT: branched-chain amino transferase; mTOR: mammalian target of rapamycin.

While our understanding of the immunosuppressive role of BCATc and BCATm in the TME is still in its infancy, accumulating body of evidence suggests that different arms of the immune system may differ in how they take advantage of BCATs and BCAA metabolism. CTLs and CD4^+^ T_H_1 cells require BCAT activity to control the increased influx of BCAAs, which in turn controls mTORC1 signaling during T-cell activation and function (Figure 7A). However, the upregulated BCAT activity in these cells may be considered immunosuppressive for CTLs when they enter the TME. This taken together with the ability of malignant cells to uptake large amounts of BCAAs and overexpress BCATc and BCATm may deplete the TME of BCAAs and contribute to impaired anti-tumor T-cell response (Figure 7B).

In contrast to T cells, macrophages are terminally differentiated and do not have the demands of rapid proliferation ^[11]^. Macrophages may use BCATs to ensure that enough energy is produced during BCAA degradation and to maintain the levels of itaconate via the fragmented TCA cycle. Itaconate is a metabolite released by macrophages during inhibition of bacterial growth. It is produced by the IRG1 enzyme, which decarboxylates the TCA intermediate cis-ataconate ^[94]^. A study by Weiss and co-authors points toward the role of itaconate in TME, as itaconate can potentiate tumor growth of peritoneal tumors ^[95]^. Because reduced BCATc expression blocks itaconate production ^[72]^, this might be an additional mechanism via which BCATc exerts an immunosuppressive role in TME.

## 7. Conclusions

This review summarized the current understanding of how the BCAAs and their degradation via BCATc and BCATm impacts T cell activation with an emphasis on the anti-tumor T-cell immunity. Among the BCAAs, leucine was given the highest consideration based on findings describing leucine as a critical activator of mTORC1 in T cells. Leucine sensing via mTORC1 signaling is essential during the early stages of T-cell activation and their lineage commitment, while leucine transamination by the BCAT isoenzymes imposes a negative feedback loop regulating leucine sensing during these processes. However, the BCAT isoenzymes appear important checkpoints during the metabolic reprogramming of other immune cells, such as macrophages. These findings all point toward a possible immunosuppressive role of the BCAT isoenzymes and identify them as targets for pharmaceutical intervention in the TME. However, the precise mechanism of how the BCAT enzymes impact the interactions between immune and tumor cells in the TME is poorly understood. This calls for more investigations, and the Ananieva’s laboratory is testing, at the preclinical level, how deletion of the genes encoding BCATc and BCATm in T cells impacts the ability of mice to fight tumor growth (Figure 7C). The generation of new pharmaceutical drugs targeting BCATc or BCATm, or the implementation of combinatorial immunotherapies with metabolic modulation of the BCAA degradation pathway, are some of the opportunities to further consider in finding efficient strategies to maintain the T-cell functionality in the TME and to address the current challenges with immunotherapeutic drug resistance.

## Author contributions

All authors participated in the design of the review manuscript. T.J.W. and E.A.A. were responsible for the writing and the final content. S.C.E. edited the manuscript and performed the bioinformatic analysis using the R2 genomics and visualization platform. The rest of the figures were made by E.A.A. All authors have read and approved the final version of the manuscript.

## Conflicts of interest

The authors declare that they have no conflicts of interest.

## Funding

This work was supported in part by grants NIH NCI 1R15CA249796-01A1—Elucidating the role of the Branched Chain Aminotransferases (BCATc and BCATm) as novel metabolic checkpoints of anti-lymphoma T cell immunity” (to EAA).
